# The effect of APOEε4 on the functional gradient of the brain default network in Alzheimer's disease and its relationship with cognitive functions

**DOI:** 10.1002/alz70856_100047

**Published:** 2025-12-24

**Authors:** Hui Zhao, Wenting Song, Haifeng Chen

**Affiliations:** ^1^ Department of Neurology, Affiliated Drum Tower Hospital, Nanjing University Medical School, Nanjing, China; ^2^ Department of Neurology, Nanjing Drum Tower Hospital, The Affiliated Hospital of Nanjing University Medical School, Nanjing, Jiangsu, China

## Abstract

**Background:**

APOEε4 allele has an increased risk of Alzheimer's disease(AD) compared to others. Functional connectivity gradients are a novel hierarchical design of functional connectivity patterns in brain networks that captures the spatial correlations between connectivity patterns in various locations. The aim of this research is how the APOE ε4 affects the functional connectivity gradients of the default mode network (DMN) in AD and how it correlates with cognitive function.

**Method:**

From September 2022 to September 2024, 271 subjects were recruited from memory clinic of Drum Tower Hospital, Nanjing University Medical School. The study included 108 healthy controls(HC) APOE ε4 non‐carriers, 25 HC APOE ε4 carriers, 81 AD APOE ε4 non‐carriers, and 57 AD APOE ε4 carriers. All individules underwent cognitive function testing, blood test of AD biomarkers and resting‐state functional magnetic resonance imaging (rs‐fMRI). A two‐factor ANOVA and Correlation tests were utilized to examine the relationship between APOE ε4, functional gradients and cognitive function in four groups. Intermediary analysis was used to investigate the relationship between the DMN functional gradient and blood AD biomarkers.

**Result:**

After controlling for sex, age, and years of education, there was an interaction between the presence of the APOE ε4 gene and the functional gradient in the right temporal lobe of DMN in two groups; In APOE ε4 non‐carriers, the DMN functional gradient was significantly higher in AD compared to HC (*p* < 0.001), while in APOE ε4 carriers it was significantly lower in AD (*p* = 0.031); Among AD subjects, APOE‐ε4 carriers showed a significant decrease in the default network functional gradient compared to non‐carriers (*p* <0.001); APOE‐ε4 carriers showed a positive correlation between DMN functional gradient in the right temporal lobe and several ognitive domains; Mediation analyses revealed that AD markers affect DMN resting‐state functional connectivity by altering DMN functional gradients.

**Conclusion:**

The APOEε4 gene impacts the functional connectivity gradient of the default network, especially in the right temporal lobe in AD patients. They are more likely than non‐carriers to experience functional network gradient derangement, which is linked to cognitive deterioration. This effect may be related to blood AD markers altering the DMN functional gradient affecting DMN resting‐state functional connectivity.

